# Rare Presentation of Bilateral Central Retinal Vein Occlusion and Leukemic Retinopathy in a Young Adult Diagnosed with T-cell Acute Lymphoblastic Leukemia

**DOI:** 10.7759/cureus.6666

**Published:** 2020-01-15

**Authors:** Joshua H Uhr, Avrey Thau, Christine Chung, Xiao Chi Zhang

**Affiliations:** 1 Ophthalmology, Wills Eye Hospital, Philadelphia, USA; 2 Ophthalmology, Sidney Kimmel Medical College, Philadelphia, USA; 3 Emergency Medicine, Thomas Jefferson University, Philadelphia, USA

**Keywords:** central retinal vein occlusion, acute lymphoblastic leukemia, young adult, emergency medicine

## Abstract

Simultaneous bilateral central retinal vein occlusion (CRVO) is a rare presentation that warrants consideration of an underlying hyperviscosity state. Increased serum viscosity can lead to the hematologic emergency of leukostasis with resultant vascular obstruction and hypoxic tissue damage. The following case demonstrates the first case of bilateral CRVO in a young adult secondary to acute lymphoblastic leukemia (ALL). A 23-year-old female presented to the emergency department (ED) with two days of worsening bilateral blurry vision and bitemporal headache. Her ocular exam was significant for bilateral intraretinal hemorrhages consistent with CRVO with chest radiograph demonstrating widened mediastinum with perihilar lymphadenopathy and serologic testing revealing ALL with blast crisis. The patient was subsequently admitted to the oncology service for induction chemotherapy. Patients with new headache and bilateral vision changes should prompt a thorough neurological and ophthalmologic exam to assess for underlying systemic pathologies. Concurrent bilateral CRVO is a rare but specific finding for systemic hyperviscosity syndrome, blood dyscrasia, polycythemia, or other serious illness. Early recognition and treatment of the underlying condition can prevent further vision loss and overall morbidity and mortality.

## Introduction

Central retinal vein occlusion (CRVO) is a common entity with an estimated worldwide prevalence of 0.8 per 1,000 individuals [1]. However, it is exceedingly rare to present in young patients [2]. CRVO risk factors include hypertension, diabetes, hyperlipidemia, and glaucoma. Retinal vein occlusion can occur in hyperviscous or leukostatic conditions such as acute leukemia, polycythemia, or thrombocytosis, resulting in hypoxic tissue damage and vision loss [3,4]. CRVO is most commonly a unilateral presentation; thus, the rare occurrence of bilateral CRVO warrants consideration of an underlying hyperviscosity state. Early recognition and management of CRVO in the setting of an acute oncologic emergency are critical preventing permanent vision loss and patient morbidity and mortality. We present a rare case of bilateral CVRO leading to a diagnosis of acute lymphoblastic leukemia (ALL) in a young female patient. To our knowledge, this is the first reported case of bilateral CRVO secondary to ALL.

## Case presentation

A 23-year-old female presented to the emergency department (ED) with two days of acutely worsening bilateral blurry vision in the setting of a constant low-grade bitemporal and occipital headache different from her usual headache. She reported nausea and vomiting for the past month and seeing "bright spots" for two weeks. She also reported decreased appetite, night sweats, dyspnea on exertion, palpitations, and anxiety over the past month and a half. Review of system was negative for fever, chills, neck pain, head trauma, diplopia, or sudden onset of headache. Her past medical history included tension headache and anxiety. Her surgical history included appendectomy, tonsillectomy, and wisdom tooth extraction. Medications included citalopram, aspirin-acetaminophen-caffeine, ibuprofen, and levonorgestrel-ethinyl estradiol. 

On arrival to the ED, the patient’s vital signs were blood pressure 161/86 mm Hg, pulse rate 114 beats/min, respiratory rate 20 breaths/min, temperature 36.8^o^C (98.3^o^F), and SaO_2_ 95% on room air. On exam, the patient was anxious but alert and oriented with an otherwise unremarkable neurological exam. Her head and neck exam were notable for a supple neck without adenopathy and moist mucous membranes. Her cardiovascular exam was notable for tachycardia and a systolic 2/6 murmur. The patient’s ocular examination was notable for visual acuity of 20/90 in the right eye and 20/50 in the left eye, with intraocular pressures 13 and 12 mmHg, respectively. Pupils were equal and reactive without an afferent pupillary defect, and extraocular movement, confrontational visual fields, and anterior slit-lamp examination were normal. Her fundus examination, performed by an ophthalmologist, demonstrated bilateral diffuse intraretinal hemorrhages in all quadrants, white-centered retinal hemorrhage and dilated and tortuous retinal vessels without disc edema, concerning for bilateral CRVO. The reminder of her exam was unremarkable.

**Figure 1:**
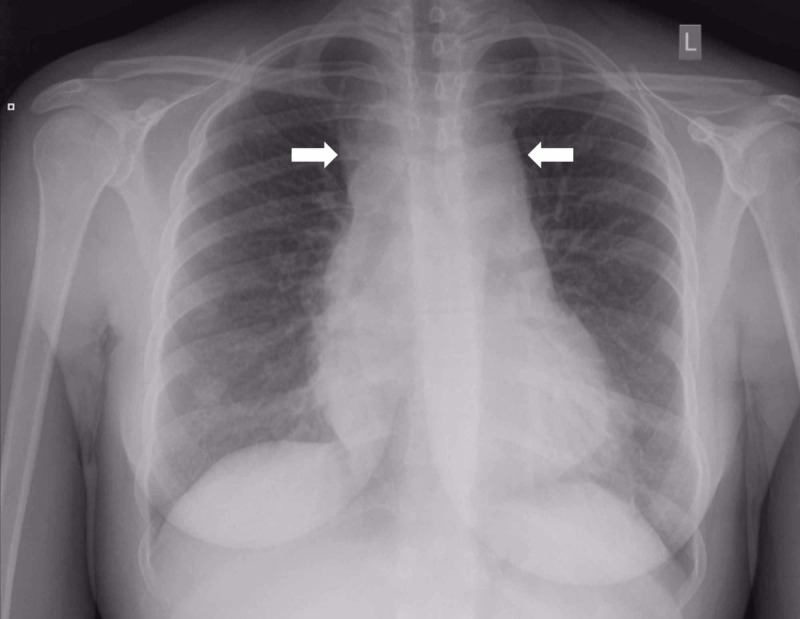
Plain film of the chest demonstrating a widening mediastinal silhouette (white arrows).

**Figure 2:**
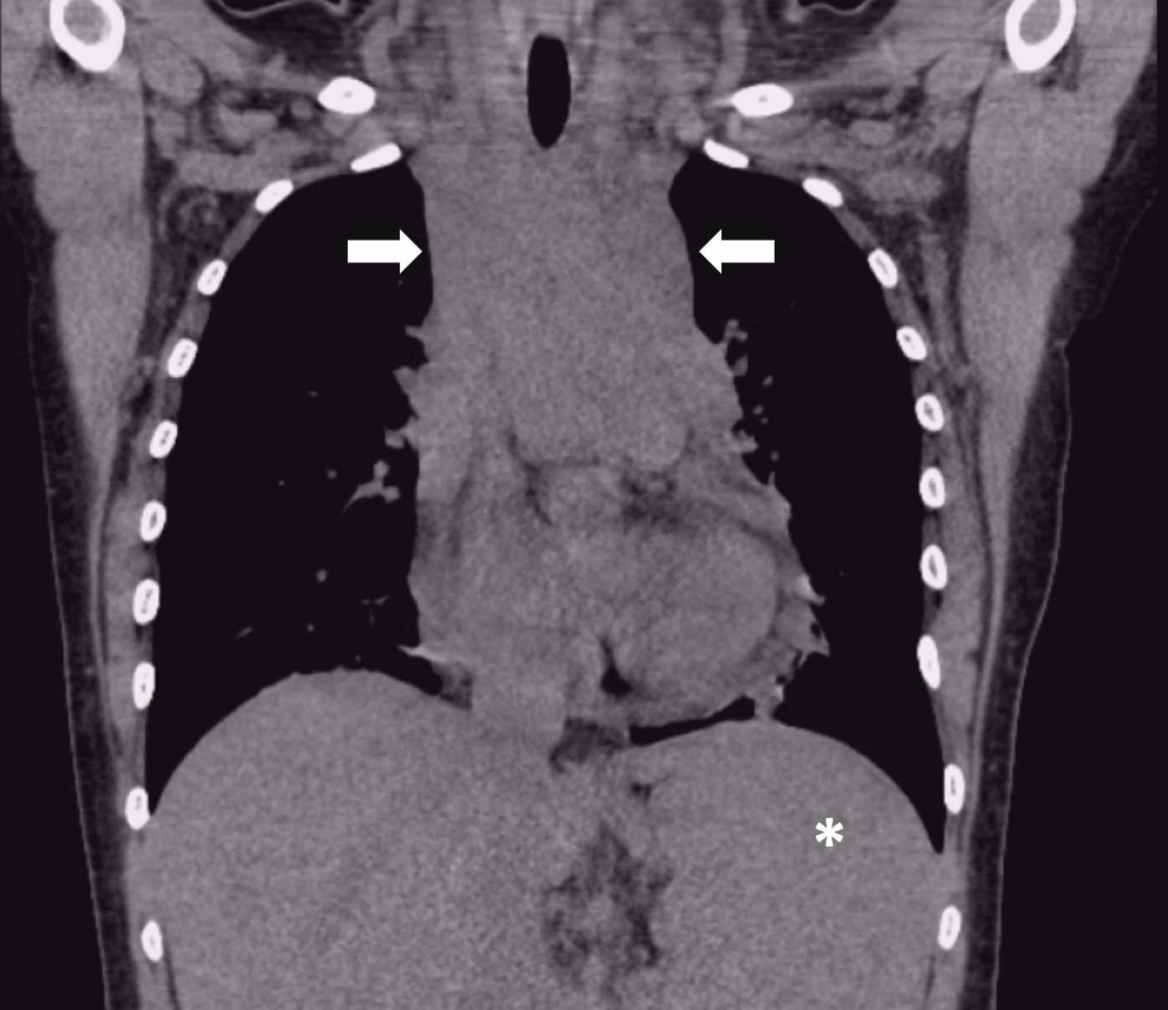
Non-contrast computed tomography with coronal view demonstrating a mass-like lobulated anterior mediastinal soft tissue density (white arrows) consistent with lymphoma and splenomegaly (asterisk).

Laboratory testing disclosed numerous chemical and serologic abnormalities with concerning findings for hyperviscosity syndrome secondary to leukocytosis (see Table 1 for details). Infectious workup for human immunodeficiency virus, hepatitis B, and hepatitis C was negative. Urine pregnancy test was negative. Chest radiograph (Figure 1) demonstrated a widening of the mediastinal silhouette with a non-contrast computed tomography (CT) of the chest demonstrating a lobulated anterior mediastinal soft tissue mass concerning for lymphoma and splenomegaly (Figure 2). Her brain CT was negative for intracranial hemorrhage or mass effect.

**Table 1:** Patient's selected laboratory studies and values

Patient’s lab test	Patient’s lab values	Reference range
Complete blood count		
White blood cell (WBC)	774 x ­10^9^/L	4-11 x 10^9^/L
Blast (absolute)	675.6 x 10^9^/L	≤0.0 x 10^9^/L
Blast (percent)	82%	≤0%
Lymphocyte (absolute)	107 x 10^9^/L	1-4 x 10^9^/L
Hemoglobin (HGB)	6.0 g/dL	12.5-15.0 g/dL
Platelet (PLT)	123 x 10^9^/L	140-400 x 10^9^/L
Reticulocyte	1.4%	0.5%-1.5%
Chemistry panel		
Potassium	5.6 mmol/L	3.3-4.8 mmol/L
Creatinine	1.6 mg/dL	0.7-1.4 mg/dL
Calcium	11.9 mg/dL	8.5-10.3 mg/dL
Alkaline phosphatase	96 IU/L	29-92 IU/L
Aspartate aminotransferase (AST)	44 IU/L	7-35 IU/L
Coagulation panel		
Prothrombin time (PT)	17.5 seconds	8.9-13.1 seconds
Partial thromboplastin time (PTT)	77 seconds	24-35 seconds
International normalized ratio (INR)	1.58	0.81-1.19
D-Dimer	1166 ng/mL	<204 ng/mL
Fibrinogen	169 mg/dL	204-462 mg/dL
Lactic acid dehydrogenase (LDH)	2187 IU/L	125-250 IU/L

The patient was diagnosed with bilateral CRVO and leukemic retinopathy with serologic and radiographic testing consistent with ALL with blast crisis, characterized by an increased number of blast cells (immature blood cells). She was admitted to the oncology service where her hospitalization stay was complicated by tumor lysis syndrome, peripherally inserted central catheter line related deep vein thrombosis, hyperglycemia secondary to aggressive steroid therapy, and transient hypotension of unclear etiology with a negative infectious workup. Her bone marrow biopsy showed T-cell acute lymphoblastic leukemia (T-ALL) with >90% marrow involved with three signals for the ABL1 gene. She was initially managed with intravenous fluids, hydroxyurea, and dexamethasone, and started treatment for T-ALL with induction chemotherapy. She was discharged after 18 days of hospitalization with prophylactic acyclovir, sulfamethoxazole/trimethoprim, and prednisone as part of her chemotherapy regimen and outpatient retina clinic follow -up.

## Discussion

CRVO may be a common occurrence for aging patients with numerous risk factors; however, it is highly unusual to present in young patients. Only 10%-15% of cases are reported to occur in patients below 40 years of age [2]. Given the rare occurrence of simultaneous bilateral CRVO in a previously healthy patient, further testing was prompted to assess for common causes for serum hyperviscosity, such as Waldenstrom’s macroglobulinemia, hyperhomocysteinemia, multiple myeloma, acute myeloid leukemia (AML), polycythemia, and chronic myeloid leukemia (CML), revealing our patient’s underlying oncologic disease process, ALL [3-9].

Retinal vein occlusions in patients with leukemia are hypothesized to be secondary to increased serum viscosity, either from leukocytosis or thrombocytosis [4]. The term “hyperleukocytosis” refers to a significantly elevated peripheral white blood cell (WBC) count of greater than 100 x10^9^/L [10,11]]. It constitutes a hematologic emergency that can lead to leukostasis, vascular obstruction, and resultant hypoxic tissue damage. Leukostasis develops more commonly in patients with AML and CML compared to patients with ALL, and tends to occur at higher WBC counts in patients with ALL (>400 x10^9^/L) [10]. This may explain why bilateral CRVO has been reported in AML and CML but not ALL. With an extremely elevated WBC of 774 x10^9^/L, this patient was at risk for complications of serum hyperviscosity. Hyperleukocytosis also places patients at risk for disseminated intravascular coagulation and tumor lysis syndrome, both of which manifested during the patient’s ED visit with elevated international normalized ratio, D-dimer, lactate dehydrogenase, prothrombin time/partial thromboplastin time, hypercalcemia, hyperkalemia, with decreased fibrinogen and platelet level [11].

Systemic abnormalities that produce retinal changes are termed leukemic retinopathy and have a diverse range of presentations [12]. In acute leukemia, common retinal findings include preretinal and retinal hemorrhages due to anemia and thrombocytopenia, while chronic leukemia more commonly presents with venous stasis due to hyperviscosity [12]. Additional reported findings in leukemic retinopathy include venous dilation and tortuosity as well as white-centered hemorrhages which may consist of leukemic cells, platelet-fibrin aggregates, or septic emboli [13]. In contrast to the diffuse retinal hemorrhages seen in CRVO, retinal hemorrhages in leukemic retinopathy are usually isolated in the posterior pole [13]. While leukemic retinopathy is more commonly recognized after the diagnosis of leukemia is established, it can represent early signs of disease or relapse [3,6,8].

Additional ophthalmic manifestations of leukemia may be the result of direct leukemic cell infiltrates, with reported expressions in nearly every ocular structure [12-15]. Ocular manifestations of leukemia are common, with studies reporting a prevalence between 20% and 90%, although its involvement is often asymptomatic [12-16]. Involvement has been more commonly cited as seen in acute rather than chronic leukemia, although this has not been consistent across all studies [12,14,17].

Unfortunately, the patient experienced a host of complications following hospital discharge and was unable to have confirmatory outpatient ophthalmologic testing performed. Her diagnosis of CRVO is therefore based on clinical examination, without confirmatory imaging such as fluorescein angiography with fundus photography and optical coherence tomography. However, based on the extent of retinal hemorrhages in all quadrants, her ocular findings were more consistent with vein occlusion than leukemic retinopathy alone.

## Conclusions

Acute oncologic emergencies can often present in numerous forms; however, delayed diagnostic and intervention due to overlooked subtle patient presentations can result in dire patient morbidity and mortality. Young patients with new headache and bilateral vision changes should prompt a thorough neurological and ophthalmologic exam to assess for underlying systemic pathologies. The clinical presentation of bilateral retinal hemorrhages is suggestive for a systemic hyperviscosity state and warrants an emergent evaluation for oncologic emergencies. In this case, bilateral CRVO in a young patient was her first presenting sign of ALL. Although it is uncommon for leukemic patients to initially present to the ED with a primary eye complaint, this case highlights that careful evaluation of ocular findings may have systemic and life-saving implications.
